# An Improved Heuristic Drift Elimination Method for Indoor Pedestrian Positioning

**DOI:** 10.3390/s18061874

**Published:** 2018-06-07

**Authors:** Zhihong Deng, Yun Cao, Pengyu Wang, Bo Wang

**Affiliations:** School of Automation, Beijing Institute of Technology, Beijing 100081, China; cy_zero@126.com (Y.C.); 18210252938@163.com (P.W.); wb1020@bit.edu.cn (B.W.)

**Keywords:** PDR, heading correction, membership function, dominant direction

## Abstract

Indoor positioning is currently a research hotspot. In recent years, Pedestrian Dead Reckoning (PDR) has been widely used in indoor positioning. However, the positioning error caused by heading drifts will accumulate as the walking distance increases, so some methods need to be used to correct the heading angle. Heuristic Drift Elimination (HDE) is an effective heading correction algorithm, which only uses the information of a building’s dominant directions to reduce the heading error, but it does not apply to the non-dominant direction condition. In this paper, we propose a heading drift suppressing method for the limitation of HDE. Firstly, the method constructs membership functions to judge the pedestrian’s motion according to the result of comprehensive evaluation. Then, it further determines by a threshold condition whether the pedestrian walks along the dominant directions, and a heading error measurement is introduced for heading correction. Finally, we verify by experiments that the proposed method can correct heading angles properly for different conditions, which indicates an adaptability to the environment.

## 1. Introduction

As technology advances, people’s lives are becoming more and more intelligent, and the demand for location services is gradually increasing. Therefore, the development of indoor positioning technology has drawn wide attention from people. It has broad application prospects in target monitoring, emergency rescue, shopping guides, and so on.

Currently, indoor positioning relies mainly on WiFi, Bluetooth, UWB (Ultra Wide Band), and other wireless location technologies, as well as optical communications, machine vision, and so forth. Using the above positioning technologies can achieve good results. For instance, when we use UWB, the positioning accuracy can even reach centimeter level. However, due to the layout of reference beacons in advance and a high cost, its application is limited. Thus, some researchers apply inertial navigation technology to indoor positioning. With the data collected by MEMS (Micro-Electro-Mechanical Systems)-integrated IMUs (Inertial Measurement Units), the pedestrian’s position can be calculated. This method can effectively resist environmental disturbance and has strong autonomy, but cumulative errors caused by MEMS devices cannot be ignored. Foxlin [1] first proposed the IEZ framework, that is, Inertial Navigation System (INS), Extended Kalman Filter (EKF), and Zero Velocity Update (ZUPT) to estimate the motion track. Some researchers carried out their work based on this method [2,3]. The framework restrains the horizontal attitude errors well, but it cannot make the heading correction. Hence, many scholars at home and abroad have conducted in-depth studies. Yang Hong et al. [4,5] adopted a geomagnetic correction algorithm to correct the heading angle by taking the difference of courses calculated by the magnetometer and attitude matrix as a measurement. Afzal [6] estimated the heading error by capturing changes in the magnetic field measured by magnetometers in the pedestrian’s stationary state. Since the magnetometer suffers more perturbations in indoor environments, the reliability of its calculated heading angle cannot be guaranteed. Therefore, some people believe that the magnetometer is not suitable for indoor use [7]. To improve the accuracy of heading calculation, Widyawan et al. [8] fused INS and visual image information, but this method was greatly affected by the light intensity. Similarly, some researchers used the particle filter method to estimate the heading angle by getting the interior plan of the building in advance [9], but most of the time it was hard to do that. Borenstein [10] proposed the Heuristic Drift Elimination (HDE) algorithm in 2010. The algorithm is based on the fact that most walls and corridors inside buildings are made up of straight lines and right angles and use only four or eight dominant directions as a reference without prior information about the environment, which can effectively reduce the heading drift.

However, as Jiménez mentioned in Reference [11], the HDE algorithm is suitable for pedestrians walking along the dominant directions, but sometimes the building does not all consist of paths along the dominant directions. Special structures, such as circular arcs, may exist, and the heading angle will be wrongly corrected if the HDE algorithm is used in this case. Thus, the algorithm needs to be improved in some ways.

In this paper, we conducted a study of the heading correction method based on the IEZ framework for the problems above. We first describe the basic IEZ framework for getting the pedestrian’s motion information, propose the improved HDE (IHDE) method, and then verify the validity of proposed method by experiments.

## 2. Pedestrian Dead Reckoning (PDR) Implementation with Kalman Filter

**Figure 1:**
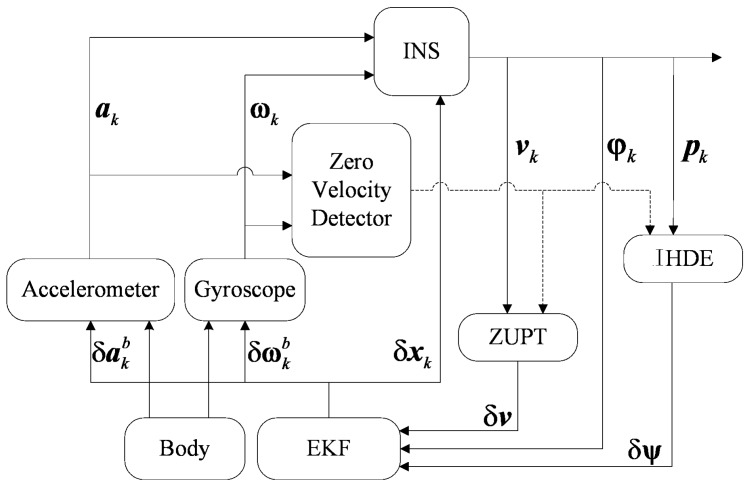
Pedestrian Dead Reckoning (PDR) implementation scheme.

PDR is implemented based on a Kalman filter. The method is to obtain the motion information by calculating the inertial sensor data. When the footfall of the pedestrian is detected, the EKF measurements will be updated by ZUPT and the estimated errors are used to correct the motion information. The 15-dimensional error state vector of EKF is $\delta x_{k}={\left[\begin{array}{cccc} \begin{array}{cc} \delta r_{k} & \delta v_{k} \end{array} & \delta \varphi_{k} & \delta a_{k}^{b} & \delta \omega_{k}^{b} \end{array}\right]}^{T}$. The vector contains the errors in position, velocity, and attitude, as well as the estimated biases for accelerometers and gyroscopes, each of which consists of its 3-axis components. *k* indicates the moment of data sampling. Figure 1 shows the PDR implementation process.

An accelerometer and gyroscope are mounted on the human body to collect data related to the motion information. The Micro-Inertial-Navigation System (MINS) block then calculates the data. The Zero Velocity Detector (ZVD) block is used to detect zero speed moments to trigger ZUPT and IHDE blocks, and these two blocks provide the EKF block with measurements to estimate errors.

### 2.1. Micro-Inertial-Navigation System

The MINS block calculates the data collected by the IMU to get the position, velocity, and attitude information. The process is divided into 4 steps:

- Data Preprocessing: Due to the drift characteristics of inertial sensors, we compensate the raw data $a_{k}$ and $\omega_{k}$ by the estimated errors $\delta a_{k}^{b}$ and $\delta \omega_{k}^{b}$ before calculating them.
- Attitude Update: We update the quaternion to get the pedestrian’s attitude information. The quaternion updating method is as follows:
  (1) $$Q_{k}=\left(\cos\frac{\Delta }{2}\cdot I_{4\times 4}+\frac{2}{\Delta }\sin\frac{\Delta }{2}\cdot \Delta \theta \right)Q_{k-1}.$$
  $Q_{k}={\left[\begin{array}{cccc} q_{0} & q_{1} & q_{2} & q_{3} \end{array}\right]}^{T}$ represents the quaternion, and $I_{4\times 4}$ is a 4 by 4 unit matrix. Since the sampling period Δt is very short,
  (2) $$\Delta \theta =\frac{1}{2}\left[\begin{array}{cccc} 0 & -\Delta \theta_{x} & -\Delta \theta_{y} & -\Delta \theta_{z}\\ \Delta \theta_{x} & 0 & \Delta \theta_{z} & -\Delta \theta_{y}\\ \Delta \theta_{y} & -\Delta \theta_{z} & 0 & \Delta \theta_{x}\\ \Delta \theta_{z} & \Delta \theta_{y} & -\Delta \theta_{x} & 0 \end{array}\right].$$
  $\Delta \theta_{i}$(i=x,y,z) indicates the angle increment of gyroscope, and $\Delta =\sqrt{{\left(\Delta \theta_{x}\right)}^{2}+{\left(\Delta \theta_{y}\right)}^{2}+{\left(\Delta \theta_{z}\right)}^{2}}$ is the modulus of angle increments. The attitude angles can then be calculated by the quaternion as:
  (3) $$\begin{array}{l} \gamma =\arctan\left(2\left(q_{2}q_{3}-q_{0}q_{1}\right)/\left(q_{3}{}^{2}-q_{2}{}^{2}-q_{1}{}^{2}+q_{0}{}^{2}\right)\right)\\ \theta =\arcsin\left(2\left(q_{0}q_{2}-q_{1}q_{3}\right)\right)\\ \psi =\arctan\left(2\left(q_{1}q_{2}+q_{0}q_{3}\right)/\left(q_{1}{}^{2}+q_{0}{}^{2}-q_{2}{}^{2}-q_{3}{}^{2}\right)\right) \end{array}.$$
  γ, θ and ψ are the roll, pitch, and yaw angle, respectively.
- Position and Velocity Update: We first transform the acceleration in the sensor body frame to the navigation frame with the attitude matrix, and then remove the gravitational acceleration:
  (4) $${\bar{a}}_{k}=C_{b}^{t}a_{k}+{\left[\begin{array}{ccc} 0 & 0 & g \end{array}\right]}^{T}.$$
  ${\bar{a}}_{k}$ represents the free acceleration, and ${\left[\begin{array}{ccc} 0 & 0 & g \end{array}\right]}^{T}$ the gravitational acceleration with *g*’s value chosen as 9.80665 m/s^2^. $C_{b}^{t}$ is the attitude matrix, where *t* refers to the navigation frame (defined to be North-East-Down on the ground) and *b* is the body frame. The pedestrian’s position and velocity are updated by the integral of ${\bar{a}}_{k}$.
- Motion Information Correction: If a zero speed moment is detected, because theoretically the pedestrian’s velocity should be zero, we take the difference between zero and $v_{k}$, that is,
  (5) $$\Delta v_{k}={\left[\begin{array}{ccc} 0 & 0 & 0 \end{array}\right]}^{T}-v_{k}$$
  as the measurements, and estimate the error state by EKF to correct the motion information. The position and velocity can be directly corrected by $\delta r_{k}$ and $\delta v_{k}$. The attitude correction is equivalent to the correction of attitude matrix:
  (6) $${\bar{C}}_{b}^{t}=\left(I_{3\times 3}-\Delta_{t}\right)C_{b}^{t}$$
  where $\Delta_{t}$ is the skew symmetric matrix for $\delta \varphi_{k}$. According to the conversion relationship between the attitude matrix and quaternion, the quaternion can then be corrected.

### 2.2. Extended Kalman Filter

● State Equation

(7) $$\delta x_{k/k-1}=F_{k/k-1}\delta x_{k-1}+G_{k/k-1}w_{k-1}$$

$\delta x_{k/k-1}$ is the predicted error state, and $\delta x_{k-1}$ is the last filtered error state. $w_{k-1}$ is the process noise with covariance matrix $Q_{k-1}=E\left(w_{k-1}w_{k-1}{}^{T}\right)$. The state transition matrix $F_{k/k-1}$ and noise driven matrix $G_{k/k-1}$ corresponding to the error state are derived from the error model of INS as follows [12]:

(8) $$\begin{array}{l} F_{k/k-1}=\left[\begin{array}{ccccc} I_{3\times 3} & I_{3\times 3}\cdot \Delta t & O_{3\times 3} & O_{3\times 3} & O_{3\times 3}\\ O_{3\times 3} & I_{3\times 3} & \Omega \cdot \Delta t & C_{b}^{t}\cdot \Delta t & O_{3\times 3}\\ O_{3\times 3} & O_{3\times 3} & I_{3\times 3} & O_{3\times 3} & -C_{b}^{t}\cdot \Delta t\\ O_{3\times 3} & O_{3\times 3} & O_{3\times 3} & I_{3\times 3} & O_{3\times 3}\\ O_{3\times 3} & O_{3\times 3} & O_{3\times 3} & O_{3\times 3} & I_{3\times 3} \end{array}\right]\\ G_{k/k-1}=\left[\begin{array}{cccc} O_{3\times 3} & O_{3\times 3} & O_{3\times 3} & O_{3\times 3}\\ C_{b}^{t}\cdot \Delta t & O_{3\times 3} & O_{3\times 3} & O_{3\times 3}\\ O_{3\times 3} & -C_{b}^{t}\cdot \Delta t & O_{3\times 3} & O_{3\times 3}\\ O_{3\times 3} & O_{3\times 3} & I_{3\times 3}\cdot \Delta t & O_{3\times 3}\\ O_{3\times 3} & O_{3\times 3} & O_{3\times 3} & I_{3\times 3}\cdot \Delta t \end{array}\right] \end{array}.$$

Ω is the skew symmetric matrix for the acceleration transformed to the navigation frame. $O_{3\times 3}$ refers to a 3 by 3 zero matrix.

● Measurement Equation

(9) $$z_{k}=H_{k}\delta x_{k/k-1}+\eta_{k}$$

where $z_{k}$ represents the measurements, and $\eta_{k}$ is the measurement noise with covariance matrix $R_{k}$ equal to $E\left(\eta_{k}\eta_{k}{}^{T}\right)$. Since the measurements are velocity errors of three axes, the measurement matrix is

(10) $$H_{k}=\left[\begin{array}{ccccc} O_{3\times 3} & I_{3\times 3} & O_{3\times 3} & O_{3\times 3} & O_{3\times 3} \end{array}\right].$$

We divide the role of EKF into two cases: if the zero speed moment is not detected, then EKF will only be updated in time, that is, only the last filtered error covariance matrix $P_{k-1}$ is updated when $z_{k}$ is obtained [13].

(11) $$P_{k/k-1}=F_{k/k-1}P_{k-1}F_{k/k-1}{}^{T}+G_{k/k-1}Q_{k-1}G_{k/k-1}{}^{T}$$

$P_{k/k-1}$ is the predicted error covariance matrix, and $P_{k}$ is equal to $P_{k/k-1}$ at this moment. In the other case, EKF is updated in state with $z_{k}$ as below:

(12) $$\delta x_{k}=\delta x_{k/k-1}+K_{k}\left(z_{k}-H_{k}\delta x_{k/k-1}\right).$$

$K_{k}$ is the filter gain.

(13) $$K_{k}=P_{k/k-1}H^{T}{\left(HP_{k/k-1}H^{T}+R_{k}\right)}^{-1}$$

### 2.3. Zero Velocity Detector

The accuracy of zero speed moment detection is closely related to the calculation result. The pedestrian’s movement can be divided into two cases: station and motion, denoted as $C_{1}$ and $C_{2}$, respectively. Specifically, the foot swings forward when moving and clings to the ground in station case [14]. We determine whether *n* is zero speed moment based on the generalized likelihood ratio (GLRT) method [15]:

(14) $$L_{G}\left(m_{n}\right)=\frac{p\left(m_{n};C_{1}\right)}{p\left(m_{n};C_{2}\right)}.$$

$L_{G}\left(m_{n}\right)$ indicates GLRT, and $m_{n}$ refers to the sampling sequence.

(15) $$m_{n}=\left\{\left[\begin{array}{c} a_{k}\\ \omega_{k} \end{array}\right]\right\}\;k=n,n+1,\ldots ,n+N-1$$

where *N* is the length of selected sliding window, and $p\left(m_{n};C_{1}\right)$ and $p\left(m_{n};C_{2}\right)$ are the probability density functions for $C_{1}$ and $C_{2}$. According to the multiplication principle in probability statistics and considering the independence of two-dimensional random variables,

(16) $$p\left(m_{n};C_{j}\right)=\prod_{k=n}^{n+N-1}p\left(a_{k};C_{j}\right)p\left(\omega_{k};C_{j}\right)\;j=1,2.$$

From Equations (14) and (16) we know, to obtain $L_{G}\left(m_{n}\right)$, it is necessary to get the probability distributions of $a_{k}$ and $\omega_{k}$ in advance, but in practice this information is unavailable. So, we represent the sample at each moment as:

(17) $$\left[\begin{array}{c} a_{k}\\ \omega_{k} \end{array}\right]=\left[\begin{array}{c} a_{k}^{r}\\ \omega_{k}^{r} \end{array}\right]+n_{k}.$$

The ideal sampling acceleration $a_{k}^{r}$ and angular velocity $\omega_{k}^{r}$ meet the following conditions: in motion case, $a_{k}^{r}$ is not equal to the gravitational acceleration ***g***, and $\omega_{k}^{r}$ is not equal to 0; comparatively, they are both equal to the ideal values in the other case. $n_{k}$ is the sampling noise. If we assume it to be zero-mean white noise with the noise variances of the accelerometer and gyroscope $\sigma_{a}{}^{2}$ and $\sigma_{\omega }{}^{2}$, respectively, then $a_{k}$ and $\omega_{k}$ follow the Gaussian distribution. Hence, based on the above equations we obtain the probability density functions as below:

(18) $$p\left(m_{n};C_{j}\right)=\frac{1}{{\left(\sqrt{2\pi }\sigma_{a}\right)}^{N}}\cdot \frac{1}{{\left(\sqrt{2\pi }\sigma_{\omega }\right)}^{N}}\cdot \exp\left(-\sum_{k=n}^{n+N-1}\left(\frac{1}{2\sigma_{a}{}^{2}}{\left\|a_{k}-a_{k}^{r}\right\|}^{2}+\frac{1}{2\sigma_{\omega }{}^{2}}{\left\|\omega_{k}-\omega_{k}^{r}\right\|}^{2}\right)\right).$$

According to the basic principle, the GLRT corresponding to moment *n* can be calculated by maximizing Equation (18) with a proper selection of $a_{k}^{r}$ and $\omega_{k}^{r}$. We substitute the maximum values for Equation (14) and reorganize the result:

(19) $$L_{G}{}^{\prime }\left(m_{n}\right)=\sum_{k=n}^{n+N-1}\left(\frac{1}{\sigma_{a}{}^{2}}{\left\|a_{k}-g\frac{{\bar{a}}_{n}}{\left\|{\bar{a}}_{n}\right\|}\right\|}^{2}+\frac{1}{\sigma_{\omega }{}^{2}}{\left\|\omega_{k}\right\|}^{2}\right)<th_{G}.$$

**Figure 2:**
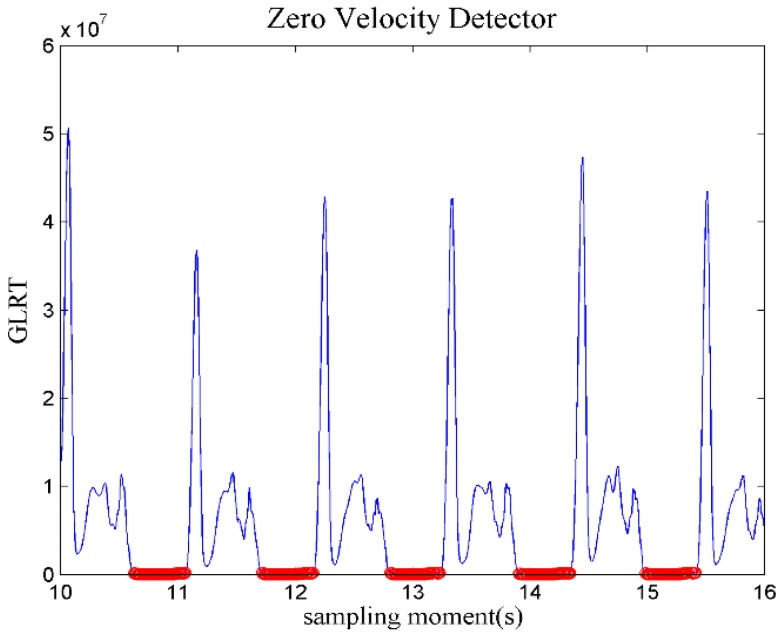
The calculated generalized likelihood ratio (GLRT) at some sampling moments.

${\bar{a}}_{n}$ represents the average value of acceleration samples in the sliding window. Through the above equation we can determine the pedestrian’s movement at moment *n*. If $L_{G}{}^{\prime }\left(m_{n}\right)$ is less than the threshold $th_{G}$, then $C_{1}$ is true, as shown in red in Figure 2, or $C_{2}$ is true otherwise.

### 2.4. The Improved HDE Method

● Limitations of original methods

Although using the IEZ framework has a significant effect upon correcting the horizontal attitude angles, it influences little on heading correction. The reason is that, according to the observability analysis of INS error equations, the error state $\delta \psi_{k}$ is unobservable [16]. Thus, the estimated trajectory will gradually deviate from the expected as the moving distance increases.

**Figure 3:**
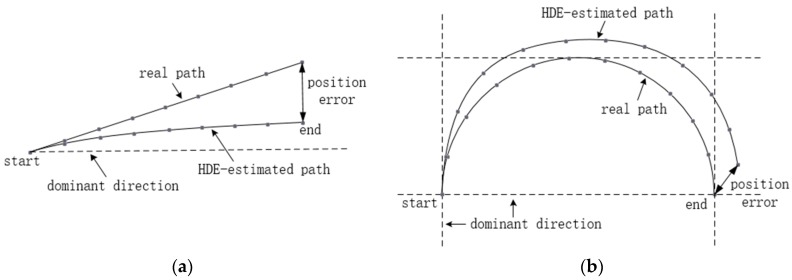
Position errors caused by the corrections of original Heuristic Drift Elimination (HDE) method for: (**a**) a straight path along a non-dominant direction; (**b**) a circular path.

In indoor environments, the HDE algorithm can effectively correct the heading angle by taking the dominant directions of the building as a reference. However, not all motion paths in indoor environments are along the dominant directions. If the HDE algorithm is still used in a non-dominant direction condition, as can be seen in Figure 3, the heading angle will be overcorrected, and a new heading error is then generated, which will lead to the wrong estimation of the motion track. Therefore, this problem needs to be considered in practice.

● Details of the proposed method

Based on the above reasons, we first determined whether the pedestrian walked in a straight line. A fixed number of successive steps are taken as the unit of calculation, and all the calculated heading angles of steps are put into a dataset. The standard deviation of the dataset is then used to determine whether the current motion path is a straight line. Considering the complexity of calculation and accuracy of results, we take *W* (*W* = 5) steps as the unit of calculation [17], and the heading angle of step *s* is calculated by MINS. The dataset of heading angles is represented as $\Psi =\left\{\psi_{l}|l=s,s-1,\ldots ,s-W+1\right\}$, whose standard deviation is obtained by the following equations:

(20) $$\{\begin{array}{l} std=\sqrt{\frac{1}{W-1}\sum_{l=s-W+1}^{s}{\left(\psi_{l}-\bar{\psi }\right)}^{2}}\\ \bar{\psi }=mean\left(\Psi \right) \end{array}.$$

$\bar{\psi }$ is the average of heading angles. As the pedestrian cannot walk strictly along the actual path, by learning from fuzzy mathematics theory, we take *std* as variable and use the Z-type membership function to describe the motion path.

(21) $$\mu_{1}\left(std\right)=\frac{1}{1+\exp\left(k_{1}\cdot std-a\right)}\;k_{1}>0,a>0$$

**Figure 4:**
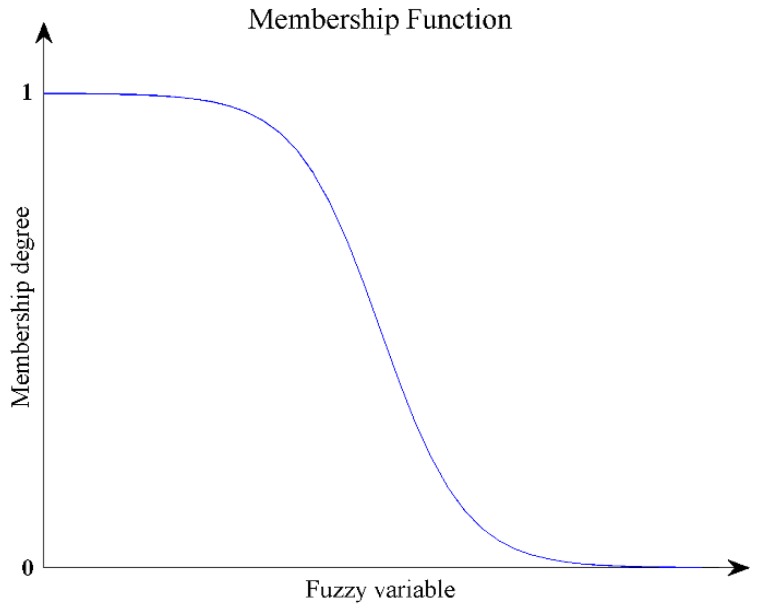
Z-type membership function.

$\mu_{1}\left(std\right)$ ranges from 0 to 1. The membership degree of motion path to the straight line decreases as *std* becomes larger. Figure 4 reflects the trend of the function.

Second, as the normal walking process of the pedestrian is periodic, the time interval between two adjacent steps, denoted as $ST_{s}$, is relatively uniform. If $ST_{s}$ is out of the normal range, the pedestrian may be in abnormal activities, and to prevent the heading angle from being wrongly corrected in this condition, the proposed method should be avoided.

Hence, we take $ST_{s}$ as variable and adopt the commonly used bell-shaped membership function to characterize the pedestrian’s motion state.

(22) $$\mu_{2}\left(ST_{s}\right)=\frac{1}{1+b{\left(ST_{s}-\gamma_{T}\right)}^{2c}}\;b>0,c>0$$

where $\gamma_{T}$ is associated with the motion period, usually set to 1.0~1.2 s. Combined with the motion path and motion state, we can make a comprehensive evaluation of the pedestrian’s motion condition and the evaluation vector *M* is ${\left[\begin{array}{cc} \mu_{1}\left(std\right) & \mu_{2}\left(ST_{s}\right) \end{array}\right]}^{T}$. Though the conditions of triggering the proposed method mainly depend on whether the motion path is a straight line, the role of the method needs to be limited in abnormal motion state. Therefore, we adopt M(.,+) model, and with the weight vector $w=\left[\begin{array}{cc} w_{1} & w_{2} \end{array}\right]$, the comprehensive evaluation is

(23) $$E=w\cdot M=w_{1}\mu_{1}\left(std\right)+w_{2}\mu_{2}\left(ST_{s}\right).$$

Then we give a binary controller as below:

(24) $$En=\{\begin{array}{cc} 1 & E>\gamma_{2}\\ 0 & else \end{array}.$$

**Figure 5:**
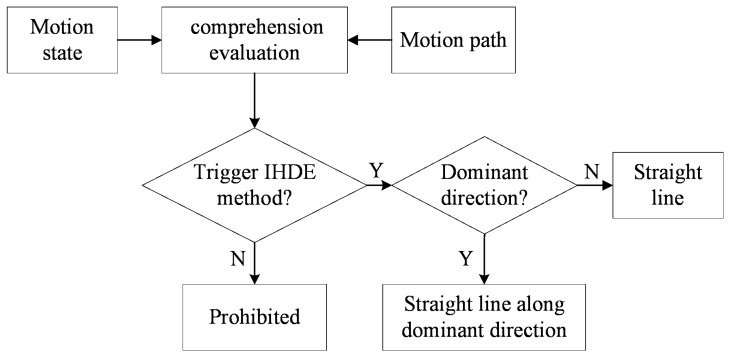
The block diagram of the proposed method.

If *E* is greater than the threshold $\gamma_{2}$, then *En* is set to 1 and the proposed method will be triggered, or the method has no effect otherwise. Figure 5 shows the analysis process above.

Finally, we get the error measurement δψ. If *En* is 0, δψ will be set to 0 as well, or δψ is obtained by determining whether the pedestrian walks along the dominant directions. We define 4 dominant directions denoted as $\psi_{d}$ (*d* = 1, 2, 3, 4) to be 0°, 90°, 180°, and 270° respectively, and select 5° and 355° as the thresholds.

(25) $$\left|\psi_{d}-\bar{\psi }\right|<5^{\circ }\|\left|\psi_{d}-\bar{\psi }\right|>355^{\circ }$$

If the above equation holds for a certain $\psi_{d}$, then it will be taken as the closest dominant direction $\psi_{dominant}$ corresponding to the current heading angle. By subtracting the current heading angle from $\psi_{dominant}$ we obtain

(26) $$\delta \psi =\psi_{dominant}-\psi_{s}.$$

If, instead, Equation (26) holds for no $\psi_{d}$, then $\psi_{dominant}$ will be substituted for $\bar{\psi }$ as the reference direction to calculate δψ [18]. After that, this error, together with the velocity errors of three axes, is taken as the filter measurements to estimate and correct the heading error, as well as other inertial sensor errors. The measurement matrix in this condition is

(27) $$H=\left[\begin{array}{ccccc} O_{3\times 3} & I_{3\times 3} & O_{3\times 3} & O_{3\times 3} & O_{3\times 3}\\ O_{1\times 3} & O_{1\times 3} & \left[\begin{array}{ccc} 0 & 0 & 1 \end{array}\right] & O_{1\times 3} & O_{1\times 3} \end{array}\right].$$

## 3. Tests

### 3.1. IMU Description

We used the IMU MTi-G-710 produced by Xsens Technologies B.V in Enschede, The Netherlands, which integrates 3-axis MEMS sensors. The IMU was easily installed on the human body, and had a large measuring range and a small size, and we fixed it to the left foot of a person to collect data. Table 1 lists the relevant technical parameters.

### 3.2. Results and Analyses

Several tests have been performed in different environments to verify the adaptability of the proposed method. For each test, we used IEZ, HDE, and IHDE methods, respectively, to estimate a person’s walking trajectories and compare results. The following index was adopted to compare the performance of different methods.

(28) $$P_{err}=\frac{D_{err}}{TTD}\times 100\%$$

$D_{err}$ indicates the deviation distance from the expected ending point, *TTD* means the total travelled distance, and $P_{err}$ is the positioning error of estimated trajectory.

**Figure 6:**
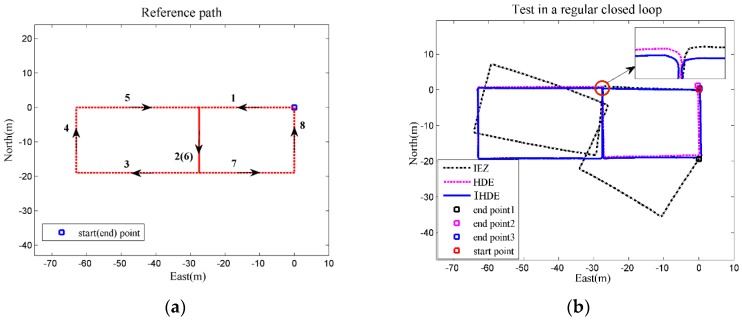
(**a**) The reference path in Test 1; (**b**) Estimated trajectories for Test 1.

Test 1 was performed on the first floor of the IT laboratory building in the Beijing Institute of Technology. The person walked from the starting point and followed the directions indicated by the arrows in Figure 6a. The 202-m long path, divided into 8 segments (identified by numbers), is made up of straight lines and right angles. Segments 1 and 7 are 27 m long, while the length of Segments 3 and 5 is 36 m, and the rest of the segments are all 19 m. Before starting to walk, the person was still for 5 s. The reference path and estimated trajectories are shown in Figure 6.

The results in Figure 6b show that, due to the lack of heading correction, the drift of the IEZ trajectory gradually accumulated in the straight lines, which led to an increasing deviation between the estimated trajectory and reference path, with the positioning error reaching about 9.0%. As each segment of the path coincided with the dominant directions, both the HDE and IHDE methods could make full use of the information to correct the heading angle. The final positioning error of the HDE trajectory was less than 0.6%, and for the IHDE trajectory, the smallest error was only 0.2% during the test.

In order to compare with the real environment, we chose the east playground for Test 2 and Test 3. Different from Test 1, the paths in Test 2 and Test 3 contained both the dominant and non-dominant directions.

**Figure 7:**
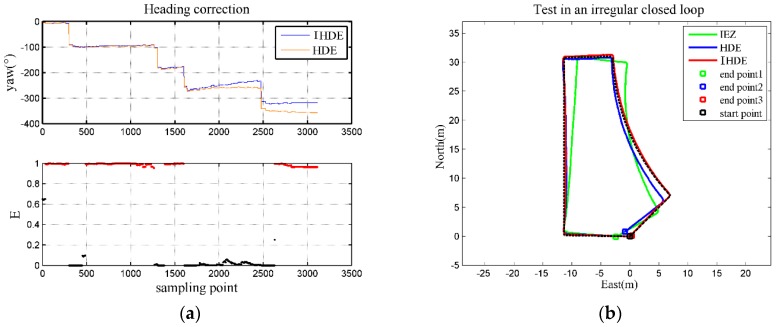
(**a**) Heading correction with IHDE; (**b**) estimated trajectories for Test 2.

In Test 2, the person first walked in straight lines along the dominant directions, then walked along the curved track for a distance, and finally walked in a straight line along a non-dominant direction and returned to the starting point. The actual path was about 90 m long, shown in a black dotted line in Figure 7, through which we illustrate the heading correction based on the proposed method. Figure 7a shows the result of evaluation E during the walk. The proposed method was triggered when E exceeded a certain threshold, corresponding to the red part, which indicated straight lines. Then, as explained in Section 2.4, we could further determine if the path was in a dominant direction and introduce error measurement δψ. It is worth noticing that between sampling point 1600 and 2475, as the person walked along a circular path, E close to 0 indicated that the method can identify abnormal situations, and thus avoid overcorrection of heading. For other parts in black, due to the sudden change of heading (corresponding to the “edges” in the above subfigure), corrections were not made within a short time.

In contrast, dominant directions were always taken as a reference in the original HDE method, so the heading was still corrected to approach its nearest dominant direction for the last two segments of path, as can be seen in the above subfigure of Figure 7a, making the trajectory wrongly estimated. The positioning error of the HDE trajectory was about 1% larger than that of the IHDE, according to the test results. Figure 7b shows the estimated trajectories using different methods.

**Figure 8:**
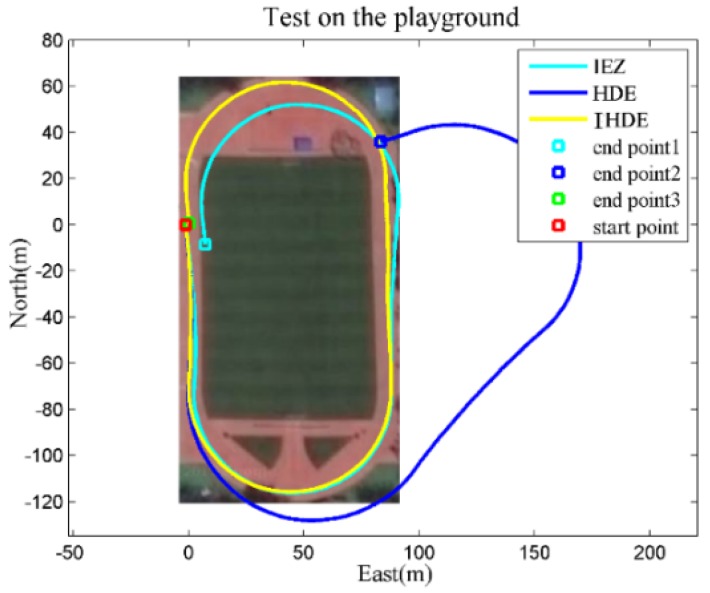
Starting somewhere on the outside straight, the person was asked to walk along the track and return to the starting point, and the entire path was approximately 455 m.

The actual path in Test 3 consisted of two straight lines along dominant directions and two arcs, as shown in Figure 8. The heading angle of the HDE trajectory was continuously wrongly corrected based on the dominant directions as a person stepped onto the curved track, which eventually led to the trajectory being useless for positioning. Instead, no heading correction was made on the curved track by using the proposed method, and the estimated trajectory maintained a small deviation from the actual path with $D_{err}$ only about 2 m. The values in Table 2 show the typical range of positioning errors for several tests in different environments like those presented in Figure 6, Figure 7 and Figure 8.

## 4. Conclusions

To solve the problem that the IEZ frame cannot suppress the heading drift, and the HDE method does not apply to the non-dominant direction’s condition, we have proposed the IHDE method and have tested its performance in different environments. The results show that, if the person walks in the non-straight line, using the proposed method can judge this condition by comprehension evaluation compared to the HDE method, and then the heading angle will not be overcorrected, ensuring the correct estimation of trajectories. The positioning errors of estimated IHDE trajectories in tests indicate the adaptability of the proposed method to different environments.

## Figures and Tables

**Table 1:** Technical parameters of MTI-G-710.

	Accelerometers	Gyroscopes
Full Scale (FS)	±15 g	±1000°/s
Non-linearity	0.03% FS	0.01% FS
Bias stability	40 μg	10°/h
Bandwidth	375 Hz	415 Hz

**Table 2:** The results of positioning errors in each test.

Method	Positioning Errors		
	Test 1	Test 2	Test 3
IEZ	8.7~9.6%	2.3~2.8%	2.6~3.2%
HDE	0.3~0.6%	1.3~1.7%	>20%
IHDE	0.2~0.4%	0.4~0.7%	0.3~0.5%
